# Editorial: Continued Fascination–A Tribute to a Giant in Immunology, Dr. William E. Paul

**DOI:** 10.3389/fimmu.2019.00354

**Published:** 2019-03-08

**Authors:** Joshua D. Milner, Jinfang Zhu, Marilyn Paul

**Affiliations:** ^1^Laboratory of Allergic Diseases, National Institute of Allergy and Infectious Diseases, National Institutes of Health, Bethesda, MD, United States; ^2^Laboratory of Immune System Biology, National Institute of Allergy and Infectious Diseases, National Institutes of Health, Bethesda, MD, United States; ^3^Independent Researcher, Washington DC, United States

**Keywords:** T cell differentiation, interleukin, William Paul, T cell, B cell

This issue honors the memory of Dr. William E. Paul, a towering figure in immunology for decades (1). Working at the Laboratory of Immunology within the NIH-NIAID for over 45 years, Bill's scientific legacy was extraordinary, matched only by the legacy of mentoring prowess (2, 3). In these pages are a series of 16 articles (15 reviews and 1 research article), most of which are authored by Bill's former trainees. It is noteworthy that Bill would often refer to those in his lab as colleagues, no matter how junior the lab member was. While this title may have felt not fitting to the trainee, there is little doubt that Bill had both a respect for those who worked for him and an expectation that one day they would have achievements worthy of making them his colleague. This gesture may well have served as one of many motivating factors which led to the extraordinary array of individuals who are Bill's legacy.

The articles in this issue cover the vast interests and expertise that germinated within Bill's lab and in the Laboratory of Immunology, and now flower throughout the scientific and medical world—validation of Bill's prescience in the title he chose for his trainees. They include the gamut of basic, translational and clinical findings covering topics which were close to home for Bill, such as the effects and regulation of IL-4 and IL-13 in various cell types (Prout et al.; Keegan et al.; Yoshimoto; Junttila), the regulation of T-cell differentiation (Zhu; Milner), T cell homeostasis (Min), mast cell biology (Brown; Caslin et al.; Huang et al.), and B cell activation (DeFranco), to those that go beyond as individual trainees followed their own unique paths (Nakanishi; Snapper). This issue also contains many important research topics that are the focuses of some independent groups (Zhu; Shevach; Kanellopoulou and Muljo; Natarajan et al.) within the Laboratory of Immunology, where Bill had served as the Lab Chief.

Below, we also include an introduction to this issue by Bill's beloved wife Marilyn, which provides all of us a more complete memory of Bill, whose endless fascination with, and contributions to the world went well-beyond science. Hopefully these articles and Mrs. Paul's personal reflections will be enlightening to the reader as the state of the art in immunology and beyond, but also provide a reminder of the enormous impact Bill has had in shaping the art, and the artists and whose work is presented here. There is no doubt that Bill's endless fascination continues.

Bill was lucky. He knew from childhood that he wanted to be a scientist. No ambivalence, no doubts. His parents wanted him to be a doctor but he wanted to be a scientist so he became both. He was lucky that he lived in Brooklyn, New York where Brooklyn College was free. His college sport was fencing. After college, he attended medical school at SUNY Downstate Medical School, also in Brooklyn. As a student, Bill lived at home with his parents. He and I married during his third year of medical school and created the home that we would share for the next 57 years. I felt so lucky that he chose me.

Although he always studied hard, he found joy in learning. He loved learning and was learned and well-read in many subjects, humanities as well as science.

Once he discovered immunology, he became devoted to it and excited by its power and potential. Immunology became his scientific home. He was proud to be a member of the generation of scientists that opened the doors of immunology to the scientific and medical academic world.

Bill loved working at the National Institutes of Health. Over the years, he had many attractive opportunities to move elsewhere. We talked it over and he stayed at NIH. He thought NIH was the best place to do basic science. He thought NIH was the best place to mentor the next generation of scientists. Bill was ever concerned that the scientific *enterprise*, his word not mine, continue.

He reveled in scientific success, his own and the achievements of others. He would come home excited and try to explain to me the importance of a recent discovery whether it was from his laboratory or elsewhere. He took special satisfaction in seeing his post docs and fellows succeed, make contributions and find jobs heading influential medical research institutions.

He was sought after as a consultant by many scientific research organizations and he thought it was important to offer his expertise to guide their programs. He wanted them to spend their money wisely. They were always surprised that he did so much work for them without being able to accept compensation. I can still picture in my mind the reams of paper he brought home to read each night until digital computer technology literally lightened the load.

In 1994, at the peak of the AIDS epidemic he took on the responsibility of becoming the Director of the Office of AIDS Research. We both understood that this was a scary time for AIDS patients and AIDS workers as well. There was a huge very ill population out there. They were scared, felt threatened and wanted a drug fast. They were angry and impatient. While working in this crisis Bill realized that more rigorous attention should be paid to vaccine research. He proposed the development of a vaccine research center for the NIH and was part of the delegation of scientists who went to the White House to convince President Bill Clinton of its importance. Clinton was persuaded. The Vaccine Research Center (VRC) opened for business in 2001. One could think of Bill as the Father of the VRC as it was his idea. I was told recently that the Building 40 of VRC went up faster than most other structures on the campus, when I attended the ceremony of dedicating the Conference Room at the VRC to Bill's memory.

Every day that he went to work was a joyful day. Every evening that he worked at home was a joyful evening. At the end of the day, he told me he quit only when he realized that he had to read something three times. He took that as a signal he was tired and should stop and have some ice cream, well-deserved ice cream.

As I stated in the beginning, Bill was lucky to find a gratifying career interest early in his life. Our sons and I were lucky too. We had the pleasure of living with a man who was so happy in his life's work. I can only hope that his life will serve as a role model for our young grandchildren. Wouldn't that be wonderful?

## Author Contributions

JM and JZ wrote the first part. MP wrote the second part.

### Conflict of Interest Statement

The authors declare that the research was conducted in the absence of any commercial or financial relationships that could be construed as a potential conflict of interest.
